# Postscript to the Historical Sketch

**Published:** 1823-07

**Authors:** 


### POSTSCRIPT TO THE HISTORICAL SKETCH.

SINCE the above Sketch was printed, we have received foreign Journals containing a variety of interesting information: from these we select some papers relative to the Nervous System, being anxious to introduce every discovery on this subject to our readers, with as little delay as possible. We have, therefore, postponed the Reviews, (although in great part prepared by the printer,) as we cannot introduce the following papers from the Journal de Physiologie with so much propriety on any future occasion; the present Number being expressly dedicated to a collection of the most important contributions to medicine made during the last six months.

In our translation of CUVIER's Report§ upon the paper of M. FLOURENS on the Nerves, we mentioned that the Editors of the Journal Complementaire referred to a work by ROLANDO, in which similar experiments were detailed. Since then we have received the Journal de Physiologie for April, from which we

§ Number for June.

take the following account of some of the discoveries made by the Italian physiologist, whose work is entitled—

ART. I.—*Saggio sopra la vera Struttura del Cervello, &c. e sopra le Fonzioni del Sistema Nervoso.* Sassari, 1809.

The short description which has been given of that part of the encephalon which is called the brain proper, will enable us to understand some experiments which serve to explain the use of this organ. The hemispheres of the brain, as we have observed, only exist in vertebrated animals, and nothing similar is met with in the inferior classes. With the intention of ascertaining what effect would result from a current of galvanic fluid passed from the brain towards the different parts of the body, I trepanned a hog, and introduced a galvanic conductor into the hemispheres, taking care to touch sometimes one point and sometimes another, whilst the other wire was applied to different parts of the body. Having repeated these experiments upon different quadrupeds and birds, I obtained only violent contractions; and I observed that these were much more powerful when the conductor penetrated into the cerebellum. The hemispheres had been torn by the repeated introduction of the point of the conductor, so that the corpora striata and ventricles were considerably injured: nevertheless the animal lived twelve hours in a comatose state, and might have survived longer had he been left unmolested.

I did not immediately deduce from these experiments the consequences at which I arrived after having discovered that the hemispheres of the brain were an assemblage of fibres, destined to produce particular movements, and after having tried on the cerebellum certain experiments, which I shall relate when I come to speak of that organ. A very active kid, on the cranium of which I applied a trepan at two different points, afforded much more satisfactory results. I introduced a stilet by one of the apertures, and divided almost all the bands of medullary matter which traverse the cineritious portion, (where it takes the name of corpus striatum.) I reached even the corpus callosum and septum lucidum; notwithstanding which, the animal remained on his feet, and walked, turning from the side which was wounded. Half an hour afterwards, I made a similar lesion in the opposite hemisphere; but I divided the filaments of which I have spoken much nearer their origin, and towards the spot where they still retain the name of crura cerebri. Although a considerable effusion of blood took place, the animal remained firm on its legs for about two hours: he did not move, except when forced to change his position by a violent shock; but neither slight irritations, noise, nor the presence of food, induced him to execute the slightest movement. After two hours he made some steps, in order to lean against the wall, or to place himself in a corner; and he passed two or three hours thus, in a state of coma or of sound sleep. Towards evening he lay down, and probably slept all night, as he was found in the same situation next morning. He was killed thirty-six hours after the performance of the experiment, in order to ascertain the parts concerned in the wound. The same experiment made upon a young lamb, afforded similar results. It is to be observed, however, that the immobility and state of drowsiness, or coma, were less remarkable here than in the kid, which is naturally more lively.

I made the same experiment on two dogs of ordinary size. Although there was an active hemorrhage in the one first operated upon, yet I introduced the stilet, first into one hemisphere and then into the other: I divided the corpora striata at different parts, I pierced the crura cerebri and the optic thalami, near the annular protuberance; after which the animal remained some time upon his feet; afterwards he lay down, and continued for ten hours in a state apparently of profound sleep, and then died, after stretching himself several times. The other dog became apoplectic on the first introduction of the stilet, which divided the corpus striatum and neighbouring parts; but, after a cut made into the optic thalami and tubercula quadrigemina, he was attacked with spasms of a tonic and atonic nature alternately: he remained for a long time cataleptic, and died convulsed some hours after.

Some very curious phenomena were likewise observed in a large hog, in which the fibres which run from the optic thalami to the corpora striata were divided with a sharp instrument. Scarcely was this done, when it was perceived that he did not move the anterior extremities as before; but it appeared that, when the animal wished to move them on one side, they moved of themselves *(spontanement)* on the other. Shortly after he fell into a profound stupor, during which he remained nearly ten hours on his feet, propped against the wall, with stertorous breathing: if he happened to get away from the wall, he immediately sought for some support. Afterwards he lay down, and, when he rose up, remained but a very short time in that position. He was killed twenty-six hours after the operation, for the purpose of examination.

Similar experiments were repeated, and varied in a thousand ways, on a great number of other animals, such as goats, sheep, &c. principally with a view to ascertain the phenomena resulting from a lesion of the tubercles, optic thalami, the corpus callosum, &c.: the results were, that as often as a great number of the fibres crossing the corpus striatum were cut or torn, that the corpus callosum or fornix was concerned, there followed a state of lethargy or stupor, and in other instances some transient symptoms of catalepsy. The appearance of stupor was always less marked in guinea-pigs, and other small animals; but I observed some other phenomena not less singular. If I removed one of the hemispheres, the animal walked and ran, turning always to the same side: on making a similar lesion on the other hemisphere, he began to turn in the opposite direction. At other times, after the same lesions, the animal ran without following any direction, knocking himself against all the obstacles which were in his way; and, lastly, using the posterior extremities as a pivot, he turned round and round, by means of the fore-legs.

I have made innumerable experiments on goats, lambs, deer, dogs, guinea-pigs, cats, and swine, to ascertain the effects of lesions of the tubercula quadrigemina, and parts in the neighbourhood of the optic thalami, but I seldom obtained the same results; which, however, ought not to excite surprise, when we reflect upon the interlacing of the numerous medullary filaments which are assembled at these parts. For, as it is extremely difficult to know what bundles of fibres have been concerned in these operations, so it is impossible to form clear and precise conclusions while the results differ. In fact, I have observed, in some of the largest of these animals, that, after having torn at one time the tubercles, at another a portion of the optic thalami, there became manifested symptoms which showed that the muscles did not move with regularity, but in a manner altogether resembling that of a person intoxicated: thus, they sometimes walked, sometimes they lifted the feet much higher than was necessary, and sometimes they trailed them along. Being unable, for many reasons, to give a detail of the observations furnished by these experiments and many others, I shall content myself with relating one of the most surprising. This I had occasion to make on some guinea-pigs, which, after I had divided the tubercula quadrigemina, and sometimes the neighbouring parts of the optic thalami, began at first to turn as usual; afterwards they lay down on one side, continually moving the limbs, principally the anterior, as if they had intended to walk. If they placed themselves on the side opposite to that on which they had fallen, they turned themselves instantly, and resumed their former position, with a promptitude resembling that of those puppets having the base made of lead and the rest of the body of some very light substance, so that they start up on one end, in whatever way they may be thrown down. If supported at the side on which they had fallen, they were enabled to walk a little; and if it happened that some began to walk after ten or fifteen days, although they might appear quite recovered, the slightest shock was sufficient to throw them over on this side, but never on the other, unless by an extraordinary effort.—Some experiments, in which the pineal gland was intersected, and even entirely separated from the peduncles,

No end to cruelties afforded not the slightest data on which a conjecture regarding its use could be formed.

*Experiments on the Brain of Birds.*—In order to understand the experiments I am about to relate, it is necessary to keep in mind what has been said above on the structure of the brain in these animals. After having trepanned the two parietal bones of a domestic fowl, with a kind of small spatula I removed from each of the hemispheres a large portion of the ceneritious substance, of which they are composed. The animal appeared at first to suffer a little; but, after twenty minutes, he began to walk, drink, and eat some crumbs of bread: he was nevertheless somewhat light-headed *(étourdi)*, as if intoxicated. When he wished to take a crumb of bread, he easily missed it, and could only catch it after pecking two or three times. I killed him twenty-four hours after the operation. On opening the brain, I found that I had removed nearly two-thirds of the substance of the hemispheres, which had been replaced by coagulated blood; but neither the medullary expansion which is situated on the internal surface of the hemispheres, nor the oblong portion which is to be found at the base, were at all affected.

Having made two openings into the parietal bones of a cock, of extraordinary vigour and activity, I removed, in the same way, a great quantity of the substance composing the hemispheres, and tore, with the same instrument, not only the above-mentioned medullary expansion, but likewise that which occupies the base of the hemispheres. I performed this operation at three separate times, leaving an interval of half an hour between each. In proportion as I went deeper in removing the parts I mentioned, the animal was more and more stupid, and remained quieter. At last he became sleepy, and lay down for some time: an hour after, he got up again, resting motionless upon his feet like a statue; and neither noise nor food, nor water, nor pricking him, could induce him to make the slightest movement: it was only a violent shock, as, for instance, pushing him with the foot, that made him change his position, and take some steps. I penetrated, with the same instrument, into the optic thalami, on each of which I made three or four incisions; which, however, afforded no new result, unless that the eyes remained open and the pupils dilated, without it being possible to make them shut by the approach of any foreign body whatever. The animal continued in this state forty-eight hours, without taking any food of himself; however, he swallowed some morsels of bread which I introduced into the gullet. I observed, after killing him at the end of this time, that the two bundles of medullary fibres, which give origin to the medullary productions above mentioned, were, as well as these last, entirely destroyed.

I repeated these experiments upon common fowls, hawks, and mallards, almost always with the same success. The effect of some lesions made on a large raven, of singular strength and sagacity *(finesse)*, are of great weight in favour of my opinion. He remained motionless like the cock, and, although he continued on his feet, he was not the less completely comatose: so much so, indeed, that he only opened his eyes when a very loud noise was made, lifting his head or resting it under his wing, as he was wont to do during natural sleep. No external object was capable of agitating him: he no longer became angry at the sight of a dog or moor-hen, his most inveterate enemies, and which he used to pursue with singular address. After he had passed twenty-eight hours in this state, I wished to make some deeper incisions; but, having inadvertently touched the part situated above the annular protuberance, he was seized with very frequent attacks of convulsions, and died in about half an hour. Having slightly touched the parts in the neighbourhood of the annular protuberance in various other fowls, it twice happened that I produced similar effects.

*Experiments on the Brains of Reptiles and Fishes.*—I had long been acquainted with the experiments of the celebrated Fontana, in which it resulted that a tortoise lived for nearly six months, continuing to eat and walk as before, after the brain had been removed. In vain did I repeat this experiment: every time that I removed the whole cerebral mass, cutting just behind the cerebellum, the animal died as quickly as those who had the head chopped off. When I had occasion to talk with this eminent individual, who cultivated every branch of science with so much success, I did not fail to question him with regard to the different result of his experiments: nevertheless, he assured me that he had always obtained the same effect in emptying the cranium entirely. I then repeated the experiment, with all the exactness possible, and causing but a very trifling effusion of blood; but every time that the medulla oblongata was much concerned, death followed; and, after twenty-four or forty-eight hours, the animal gave no sign of mobility, even under the action of galvanism.

Seeing that all these experiments were unsuccessful, I tried to vary them, and with this intention I removed only the two hemispheres of the brain of a tortoise, leaving the other parts untouched. It lived for a very long time, as did many others operated upon in a similar manner. After this ablation of the hemispheres, these animals became more stupid: they did not lose, it is true, the faculty of moving themselves, but they did so rarely, and only when they were strongly irritated. I removed the optic thalami in some others: the result was, that they only appeared a little more stupid. I allowed one of these to live two months: his death was occasioned at that time by the *musca carnaria* having deposited its larvæ in the cavity of the cranium, and these, being developed, probably destroyed the rest of the cerebral mass.

I cut and removed the two hemispheres of the brain of a very large turtle, in which this operation presents more difficulty, on account of the great mass of muscles which envelop the bones of the head. Having placed her in water again, she swam for some time, and then went to the bottom, where she remained tranquil and without moving for hours together, only turning sometimes to one side and sometimes to the other. However, when raised by means of a cord to which she was attached, she again swam a little, and then a second time let herself drop to the bottom of the water.

I removed the two hemispheres of the brain of the *squalus catulus* of Linnæus, and, having replaced him in the water, he fled with the greatest precipitation, although he had the belly pierced with the hook with which he had been caught; he hid himself behind a stone, and did not move unless he was disturbed.—I repeated and varied these experiments upon tortoises in a thousand ways, and the result was always the same.

As we can no longer distinguish true hemispheres in animals without vertebræ, it was not possible for me to make upon these last any experiments of the same nature as those related above. I shall have occasion to mention some in speaking of the cerebellum. I would only observe at present, that very slight lesions of the parts which occupy the place of brain in the *sepia*, or of the ganglions of the *lapsia*, and others of the molluscous or crustaceous tribes, no ways affect their functions; but, if these parts be more severely treated, the animal dies in a very short time.

*Experiments on the Cerebellum of Mammiferous Animals.*—The structure of the cerebellum, and the important discoveries made by the Professor of Padua, with regard to the great number of leaves *(feuillets)* of which it is composed, led me to conjecture the true uses of this organ. I believed that it was destined for locomotion, and, to confirm this opinion, I undertook the following experiments.—I made an opening, by means of a trepan, on one side of the cerebellum, and removed at different times as much of it as I could, in some swine and a sheep; but the lesion scarcely extended beyond the side which was operated upon, when the animal was struck with hemiplegia, and soon died amid convulsive spasms and hemorrhage. It is extremely difficult to penetrate into the cerebellum of quadrupeds without immediately depriving them of life; and the kid is, of all others, the animal which has appeared to me the most proper for this experiment. Having made an opening with the trepan, I cut in different directions, with a sharp stilet, the cerebellum of one of these; after which, he could no longer support himself on his legs, but seemed to be paralysed. He lived twenty-four hours in this state, and then died in convulsions. Having examined the head, I found, besides the lesions of which I have spoken, a great quantity of coagulated blood in the fourth ventricle: I imagine this was the principal cause of the spasms and death.

I should fail in the conciseness which I am anxious to observe, was I to relate minutely the experiments which I have multiplied, in various ways, on the cerebellum of a great number of quadrupeds: I shall content myself with observing, at present, that I have always found the loss of motion in direct proportion to the injury done to the cerebellum. This is the reason why the animal was sometimes entirely paralytic, and sometimes on one side only; at other times the anterior or posterior extremities alone were motionless, according as the organ was more or less completely destroyed.

*Experiments on the Cerebellum of Birds.*—I trepanned many species of birds at the point corresponding sometimes to the upper, and sometimes to the lateral part of the cerebellum; and the power over the muscles of locomotion always diminished in proportion to the lesion inflicted. Having effected an opening at the upper part of the cerebellum of a cock, by means of an appropriate instrument, I removed about one-half of this organ on the right side: on the instant, he was struck with palsy, and fell on the same side, without the power of assisting himself in the least with his right leg, or performing the slightest movement with this limb. To ascertain this singular phenomenon still further, I took the limb of the wounded side in my hand, and, by holding it in a favourable position, the cock supported himself with the other leg, and could take some steps; but shortly after he could not make any use of it, and the paralysis at length extended to both sides.—It is to be observed, that, during these alterations of the cerebellum, the animal becomes stupid and drowsy: he retains the eyes open, and looks at different objects; but it is in vain he attempts to execute the slightest movement by means of the muscles which serve for locomotion. It must be confessed, however, that he sometimes shakes his wings, and that he likewise moves the legs; but these movements seem merely to result from the mobility which the muscular fibre still retains; or else they are produced by some considerable portion of the cerebellum remaining untouched, so that it can still in part perform its functions. If it was torn at once, or the entire organ removed, the animal was constantly seized with complete palsy; but, when the lesion was only slight, he recovered the faculty of moving after a few hours.

*Experiments on the Cerebellum of Reptiles and Fishes.*—The experiments which I made on cold-blooded animals afforded similar results. A tortoise, in which I separated the cerebellum from the medulla oblongata, remained completely paralised, and lived for ten or twelve days without having made the slightest movement. After such an operation, another tortoise lived for two months, sensible as before to the slightest offence and the least stimulus, but motionless to such an extent as to be unable to remove itself by any means on being incommoded. I treated a lizard in a similar manner, and with similar result; but, what is singular, I found the same phenomena occur in two serpents, of an extremely lively kind, *(coluber natrix.)* Not having entirely removed the organ on which locomotion depends in the first, (which was the smallest,) he remained paralytic during two or three hours; but in the end he recovered his former strength, and made his escape. The second, being better managed, was entirely deprived of the power of motion; only from time to time he was aggravated by irregular movements, which were not directed by the instinct, but depended upon the great mobility of the muscular fibre in these animals. He died at the end of five days.

In order to render this experiment complete with regard to fishes, which naturally die if they are kept out of their proper element, I fixed a fish, *(il pagello,)* weighing about two pounds, with strings to a little table, and retaining him thus immersed in water, I removed the whole of the cerebellum: having then set him at liberty, he fell as if dead to the bottom of the trough, although afterwards he lived. I performed the same experiment on the *squalus catulus*, with much more facility, because the cranium of this fish is cartilaginous, and because he can remain longer out of water. He lost the power of moving himself; and, having replaced him in the water, he only made some vague and irregular movements, and could no longer swim.

I observed, as already mentioned, that the wound of the cerebellum of many fowls were quickly healed, and that these animals recovered their usual facility of movement. Nevertheless, this happened in a still more singular manner in the first tortoise I operated upon, and in which I had torn and divided the organ. The animal remained paralytic for many hours, but afterwards he quickly acquired a surprising alertness,—so much so, that he walked with a rapidity, I might say, four times greater than before. I was anxious to examine the cerebellum, which was only covered with coagulated blood; it appeared cicatrised, and considerably increased in volume.—Is it possible that the cerebellum, having acquired, by means of the cicatrization, a greater development, was thus able to contribute to the unwonted agility which the animal possessed after the operation?

*Experiments tried upon non-Vertebrated Animals.*—I extended these experiments to animals without vertebræ, in which it is impossible to say, with certainty, what part performs the function of the cerebellum; the encephalon being composed of two or more ganglions, situated about the œsophagus. It is likewise difficult to remove one part of the ganglions which compose the brain of the mollusci, without bringing the life of the animal into great danger. Of these animals, the one which appears to me most proper for this operation is the *laplisia*, in which the three principal ganglions surrounding the œsophagus form the centre, from whence arise all the nervous filaments distributed over the body. I cut these dexterously about the middle, leaving them still connected. The animal did not appear to suffer from any severe or sudden injury; which, however, was the case if two entire ganglions were removed, this last operation speedily proving fatal.

The animals in which the nervous system is interspersed with numerous ganglions bear, with less evil, the infliction of severe wounds made upon the parts corresponding to the brain and cerebellum; such are insects and the crustacei. No one is ignorant that we may cut off the head of a fly, a grasshopper, a beetle, &c. without the animal being entirely deprived of locomotion; because the numerous ganglions may serve the purpose of the cerebellum to the parts receiving nerves from them. I have observed that the locomotion is performed in a much less perfect manner, and that none of these insects live more than twenty-four or forty-eight hours after they have been decapitated. Lastly, they remain deprived of all motion, if, in separating the head with violence from the trunk, the whole nervous cord be torn, or otherwise implicated; the contrary being the case, if care be taken, in cutting off the head, that the ganglion situated under the œsophagus be left entire.

From all I have said, I think we may conclude that the organs on which sensation and motion depend are so blended and united in non-vertebrated animals, that it appears to me impossible to separate them; an assertion which I purpose illustrating more fully, until I shall have an opportunity of showing that the sensitive functions in these animals are so limited, that they scarcely have occasion for a particular organ to bring them into exercise."

Such is an extract of that part of ROLANDO's work which relates to his experiments; and, in the Journal before us, a note is given by M. MAJENDIE, both at the beginning and end of the paper, the object of which is to exculpate M. FLOURENS from the charge of plagiarism. This charge has been made in direct terms by M. COSTER, a pupil of Rolando's, in the "Archives Generales de Medecine." The defence set up by Majendie is, that the conclusions drawn by M. Flourens are very different from those of Rolando; the latter regarding the cerebellum as the source and origin of all the movements, while the former holds it to be the regulator and balancer of them. According to this view, after the removal of the cerebellum, the motions exist, and may be very energetic, but no longer co-operate in walking, flying, &c. In short, it is maintained that a great difference exists between the function of *originating* and *regulating*. Notwithstanding all this, Majendie is obliged to confess that there is a " singular analogy" between the experiments lately performed in France, and those performed in Italy twelve years ago; but he is far from drawing any conclusion from this circumstance unfavourable to M. Flourens: on the contrary, he informs us that he himself has *discovered* what had been known before. This appears to be in allusion to the curious investigations lately prosecuted with so much zeal, with regard to the uses of the anterior and posterior bundles composing the spinal nerves; and M. Majendie seems to have a fellow-feeling for M. Flourens, having himself been fain to give up the merit of priority to Mr. Charles Bell. The French have a most unfortunate propensity to claim every discovery and improvement as their own, and we must confess ourselves uncharitable enough to view the claims of Flourens with considerable distrust. Our readers will find the Report on M. Flourens' paper in our last Number, which we preferred as more clear and instructive than his own account; and, by comparing it with the one given above, they will see that M. Majendie might well term the resemblance between the experiments of the Italian and French physiologist "singular;" for, in fact, they are in many respects identically the same:—such, for instance, are the experiments on the hemispheres, producing a state analogous to sleep. There is this difference, however, in our opinion, between the papers,—that by M. Rolando gives the result of a much greater number of experiments, on a greater variety of animals, much more clearly and satisfactorily detailed, without any of those attempted refinements, and that affectation of language, which would detract considerably from the interest of M. Flourens' observations, even if we leave him the merit of originality. The subject itself is one of great interest, and M. Flourens has conferred an essential obligation on the profession by reviving it. The experiments of both physiologists on the cerebellum appear mutually to give and receive strength from the observations of M. Wenzel. We trust our readers have not forgotten that, in reviewing Dr. Cooke's work, we mentioned that that distinguished pathologist had found the cerebellum more or less diseased in every one of nineteen persons examined by him, who had laboured under epilepsy; affording a strong presumptive proof, either that it is the origin of voluntary motion, according to Rolando, and, being impaired, ceases to supply the motive power with regularity; or that it is the governor of the different movements, and loses its command over them when diseased, according to the doctrines of M. Flourens.

### Art. II.—*Remarks on the Functions of the Spinal Cord.* By M. Majendie.

The essays which I have published on the functions of the spinal nerves, which demonstrate that the anterior ones are intended for motion, and the posterior for sensibility, naturally induces a wish to examine whether the anterior and posterior sides of the spinal marrow do not possess the same property. Facts here confirm conjecture. If any portion of the spinal marrow is exposed, and touched or pricked posteriorly between the roots of the nerves, the animal evinces signs of an exquisite sensibility: if, on the contrary, the same experiments be made on the anterior part, the indications of sensibility are scarcely perceptible. The same circumstance occurs in the centre of the cord; which may be touched, nay almost torn, provided care be taken to avoid the surrounding medullary substance. I have often introduced a stilet, for a considerable distance, in the centre of the spinal marrow, without any apparent diminution of the animal's sensibility or power of motion.

In general, the properties of the spinal marrow appear to have their seat on its surface. This is evident, at least, with regard to sensibility; for, however slightly the posterior filaments are excited, even when covered by vascular membranes, acute pain is produced; and it is worthy of notice, that a decided contraction takes place in the muscles which receive their nerves from below the part touched. The contractions only occur on the side of the spinal column which is irritated.

It would be desirable to know how sensibility and motion are propagated from the medullary substance of the brain. Anatomical disposition indicates that sensation is more particularly directed towards the cerebellum, and motion towards the cerebrum; but anatomy is not sufficient, unless physiological and pathological facts assist in confirming this supposition: at present, neither the one nor the other have established what anatomy appears to demonstrate so evidently.* Lesion of the cerebellum does not destroy sensibility: abstraction of the hemispheres does not necessarily produce loss of motion. The contrary assertion, made by M. Rolando, is not correct: this physician appears to have been deceived by an accidental circumstance.

When the hemispheres are totally removed, an effusion of blood takes place, which, filling the cavity of the cranium and becoming coagulated, compresses the spinal marrow, and produces the state of torpor noticed by M. Rolando. But, if the formation of this coagulated mass be

* At least, if we regard the descriptions given by the most recent authors. It appears to me, however, that some facts might be added to the anatomical history of the junction of the spinal marrow with the brain and cerebellum. I have commenced some inquiries upon this point, which has now become of more interest than formerly.—M.

We confess ourselves unable to follow M. Majendie in his remarks on this point, being quite unable to understand what the *anatomical* facts are that would induce us to refer sensibility to the cerebellum, and motion to the brain: on the contrary, we believe that the *physiological* facts related by Rolando and Flourens, as well as the pathological observations of M. Wenzel, go to prove the reverse of this.—Editors.

prevented, the symptoms are quite different: the animals are in a state of continual agitation; they run or fly with surprising agility, unless too much weakened by loss of blood. The animals on which this experiment succeeds best, are rabbits, a month or six weeks old, or young jays and magpies that have begun to eat alone. It is curious to see them spontaneously running, jumping, &c. after the complete removal of those parts of the brain placed a little anterior to the optic tubercles. But, if the section be made immediately before the latter eminences, every thing is stopped; the animal falls on its side, the head is thrown backwards, the legs become rigid and impelled forward. I have seen young rabbits remain many hours in this position. A section made behind the optic tubercles is sufficient to remove this state; the extremities immediately lose their rigidity, and the head is again brought forward. It appears from these facts, that the corpora quadrigemina, optic thalami, and crura cerebri, perform the functions relative to motion; and it becomes necessary to examine them under this new point of view. The effects of a partial or total removal of the cerebellum is more difficult to observe, on account of the hemorrhage which always accompanies an incision in this organ, the effusion which inevitably takes place, and the compression of the spinal marrow. From many experiments, I am induced to believe that sensibility is not lost: besides, the experiments of Lorry, Legallois, &c. &c. have demonstrated that this property is inherent in the spinal marrow. It is to be hoped that this obstacle will speedily be removed, as many very zealous persons are at present exerting themselves in making researches on this point. I myself am making every effort to arrive at some satisfactory conclusions on this important subject.

I have hitherto constantly remarked, that the cerebellum appears to be absolutely necessary to the integrity of straight-forward motion; any serious injury to that organ rendering that kind of progression im possible, and motion then appearing to be effected in a retrograde manner. A duck, the greatest part of whose cerebellum I had removed, swam backwards, and made no other kind of progression for about eight days.

The following fact appears to me one of the most important respecting the functions of the anterior and posterior fasciculi of the spinal marrow, and which assists in confirming what I have advanced on the use of these parts. I am indebted for it to the kindness of M. Professor Royez-Collard.

An individual, named Sprévale, an ex-fusilier in the fifth brigade of Veterans, was admitted into the Maison de Santé, October 17th, 1806, and died March 3d, 1823. No information could be obtained as to his state of health previous to his admission. During the first ten years of his residence, he was observed to be of a taciturn and slothful disposition; never happy but in bed, and scarcely answering questions put to him; his gait unsteady, the inferior extremities weak, the superior unaffected; his pulse weak and slow. Sometimes he sallied from his apartment, and evinced a churlish disposition, and inclination to strike any one near him. The affections of the legs rapidly increasing, he was at last unable to walk. During nearly seven years from this period, he remained with the legs completely doubled up, the heels coming in contact with the pelvis, never making any use of these parts, which, however, still retained a portion of their sensibility. He could understand any thing said to him, but his answers were indistinctly articulated. His intellectual faculties nearly annihilated, he lived merely to eat, drink, and occasionally put himself in a passion. His excretions were passed involuntarily. Three weeks before his death, he was seized with a violent diarrhœa, which increased till his dissolution; the pulse was nearly imperceptible, and emaciation extreme, the trochanters and perineum being excoriated.

*Necroscopic examination.*—The tables of the cranium were three times their natural thickness; the dura mater thickened, but not injected; the arachnoid perfectly natural. The pia mater of the brain offered nothing remarkable; *but that part which covers the olivary and pyramidal eminences, as well as the anterior surface of the spinal marrow, was observed to be very dense, of a bluish colour, and dotted. This discolouration was limited on each side by the anterior roots of the nerves, and by the ligamentum dentatum*, and above it gradually diminished, on the commissure of the cerebellum, at the upper margin of which it regained its natural appearance; below, it ended with the spinal marrow. This membrane being removed, the olivary and pyramidal bodies were as soft as pulp, and of a greyish colour: this softening was continued along the whole of the anterior part of the marrow, diminishing gradually, and in almost the entire thickness of the bundles of fibres which form it. Towards the encephalon, it might be followed across the commissure of the cerebellum, into the crura cerebri, the optic thalami, the corpora striata, and some of the convolutions of the brain, particularly towards the middle of the right lobe. The anterior roots of the spinal nerves might be distinguished on the fasciculi which form them, but had not their usual consistence. All the other parts of the brain, as well as the cerebellum, were in a natural state, with the exception of the commissure of the latter; which, being of unusual firmness, strongly contrasted with the softness of the surrounding parts. *The posterior surface of the spinal marrow, and its membranes, were in a healthy state.*

Nothing remarkable appeared in the chest. In the abdomen, a small quantity of effused fluid was observed, and a few red spots on the peritoneum; the internal membrane of the stomach was of a bluish cast, spotted throughout its whole extent; that of the intestines exhibited likewise some red spots. The inferior extremities could not be extended, (thirty hours after death, the upper extremities were relaxed;) but were rendered pliable by cutting across the flexor muscles. A large quantity of synovia was contained in all the joints.

The bones, with the exception of those of the cranium, were in a natural state. It might have been thought that the increased density of the latter was occasioned by an excess of salt contained in them: experiments proved this was not the case.—100 parts, according to the analysis of M. Lassaigne, contained, animal matter, 42; phosphate of lime, 46; carbonate of lime, 12.—100 parts of the same portion of bone from a healthy subject, of the same age, yielded, animal matter, 41; phosphate of lime, 47; carbonate of lime, 12.

Does not this observation tend to prove the distinct properties of the anterior and posterior parts of the spinal column? It is proper, however, to state that the arms retained some power of motion. This latter circumstance shows the necessity of making fresh examinations of the anatomy of the spinal marrow, and its vital phenomena. This part is much more complicated at its upper extremity than any where else; and nothing is yet known as to the uses of the corpora olivaria and pyramidatia, either anterior or posterior.

---

Art. III.—*Destruction of a great Part of the Spinal Marrow, with Contraction of the Arms, and perfect Mobility of the Lower Extremities.** Case related by R. Rullier, M.D.

M. L., the subject of this observation, was forty-five years of age; his stature was short, his body slender, and his temperament highly nervous. He had been for some years receiver of the lotteries at Paris. Born in Bresse, of healthy parents, this patient had a sister affected, from childhood, with a singular nervous complaint, and which had gradually brought her to a state of idiotcy.

His intellectual faculties were developed at an age remarkably early. We are assured that, at three years old, he not only conld read, but that he could read aloud very agreeably to a numerous company. He could also write from his tenderest infancy; and a slight distortion, which appeared in the dorsal portion of the vertebral column, was attributed to this premature study of writing, having produced an elevation of the right shoulder. This ricketty disposition continued; but it had nothing very alarming about it during the youth of the patient.

The education of M. L. was very carefully attended to. He applied himself successfully to the study of languages, of which he mastered a considerable number. He cultivated, with equal success, mathematics and the belles lettres. His imagination was lively, his wit extremely keen, and eminently satirical. Left to his own direction at an early age, M. L., endowed with an ardent temperament, gave himself up without restraint to the commerce of the sex; and in this respect indulged, nearly the whole of his life, in the greatest excesses. Often reduced, by this conduct, to a state of great exhaustion, slight intervals of repose had been alone sufficient to re-establish his strength. His health, commonly very good, was seldom troubled but by some slight gonorrhœas; which, however, his intemperance kept up for a length of time.

Active and in good health, leading a life of continual pleasure, M. L. arrived at the age of thirty-four without any remarkable accident; but he then began to experience some trouble in the motions of his arms. It soon became necessary for him to have recourse to assistance in putting on his coat, and he was not long before he felt pain and some degree of swelling in that portion of the vertebral column which was distorted.

* The case we are about to describe is one of the most remarkable with which pathological anatomy has furnished us: it shows how far we are yet from knowing all the functions of the nervous system; and in this point of view it may be of great use.

This indisposition, which at first was subject to long intervals of remission, all on a sudden made a rapid progress, and the patient suddenly lost the use of his arms. It was on the 21st January, 1815, that, having gone to take a walk to St. Denis, he fell down by accident, with his face to the ground, and remained in that posture, his arms being of no use whatever in making the attempt to get up again, and which he was not able to do without assistance. His arms, since that time stiff, crooked, and involuntarily contracted, are turned so that the palms look outwards and backwards.

Since this fall, the unhappy patient, absolutely powerless in both his arms, did not enjoy one moment's health. He suffered much from the distortion of the spine, which had increased sensibility; the shoulders, especially on the right side, became raised, and the head appeared sunk between them. The patient, who in walking could not prevent the swinging of his arms, presented the appearance at once of a hunchback and a scaramouch *(pantin.)*

Uneasy, and justly alarmed at his situation, M. L. consulted the most distinguished medical men of Paris: they all believed that he was suffering under curvature of the spine *(maladie de Pott;)*—that is, a softening and caries of the vertebræ of the back, and, consequently, pressure on the spinal marrow. The treatment was, therefore, directed according to this view, and continued with great perseverance; but neither numerous blisters, nor cauteries, nor moxas, applied along the vertebral column,* and especially in the neighbourhood of the tumor, produced any relief. A crowd of other remedies, both external and internal, administered for seven successive years, had no better effect.

It was on the 5th October, 1822, that I was first called to visit M. L. then in the most deplorable state, and arrived at the most extreme degree of exhaustion and marasmus. The following were the symptoms that presented themselves:—The countenance was that of a ricketty person,—it was thin, expressive of pain, and flushed; the size of the cranium and head formed a strong contrast with the smallness of the body; the neck and limbs were long and thin; the shoulders elevated and brought forwards, approaching the ears; and the vertebral column, evidently distorted, formed a slight incurvation in the upper and right side of the region of the back. Excepting the arms, including from the shoulders to the hands, all the parts preserved their powers of motion. The patient stood upright, and walked. He was able, indeed, even a short time before his death, to go out without fatigue, and to be led, on several occasions, to the exterior Boulevards, where he took a short walk. Sustained by his nervous activity, he assured me that he felt himself strong. The arms of this patient were entirely stiff and contracted permanently, often painful, and always very troublesome; they were turned inwards, and as it were applied to the sides, from whence they could not be removed without a certain degree of effort. The fore-arm was in a state of pronation, and the hands bent. All the fingers were crooked; and, in his sleep, the nails would have injured the skin of the palm, if he had not taken the precaution, before going to

* It is not said whether absolute rest was enjoined or not.—Editors.

rest, to put his hands one within the other, in such a way that the fingers mutually opposed the excess of their action. It was in this position that the patient kept his hands when awake, both when sitting up or lying down. The want of power in the arms was complete: nevertheless, with effort, and assisted in a singular manner, a pen being put between his fingers, M. L. was enabled to make his signature, by a sort of movement of locomotion, nearly of the whole arm. This contraction appeared particularly to belong to the upper limbs ; it extended, however, to the muscles destined to move the arms towards the chest, and especially to the pectoralis major and minor. We could not decide exactly what degree of influence an analogous condition of the intrinsic muscles of the walls of the thorax had upon his habitual difficulty of breathing, and the threatenings of suffocation which tormented him, and which, an hundred times in a day, disturbed his repose or interrupted his sleep.

The condition of the arms placed this unfortunate patient in a state of the most complete dependence upon those who had the care of him. When he was lying down, he could not perform the slightest motion of the trunk of the body : thus his miserable existence was passed in intreating a change of posture,—that he might be placed upright, or assisted out of his bed. He could not bear, without extreme pain, that the same point of the chest, the back, or the sides, should support the weight of his body for any length of time. The contracted parts preserved their sensibility to the touch. The hands only ceased to serve this purpose, because they could not move; but they were, as well as the rest of the limb, sensible to the difference of temperature, and to the slightest contact. When he was touched, the patient begged that it might be done with caution, because every sudden or continued movement, given to the diseased parts, produced pain.

M. L. bore his unfortunate situation with great impatience. His moral and intellectual faculties were in full force. His sleep was most painful, and perpetually interrupted by painful stitches, which were propagated from those parts of the body that sustained its weight, towards the chest, where they produced violent palpitations of the heart, and imminent danger of suffocation. The patient was also, upon awakening, menaced as it were with apoplexy; and he did not recover from this painful state until he was placed in a sitting posture, or until he left his bed to walk about the room.

The patient coughed and spat for some months; the expectoration, tough and white, having all the external characters of cream, was produced with great difficulty. The arms contributed nothing to the expectoration; and the parietes of the thorax only preserved in part the freedom of their movements.

M. L., tormented besides by hectic fever, suffered frequent and irregular accessions and intermissions of this symptom. His nights passed usually in the most cruel torments: in the day, he was better. He felt himself relieved by a vertical position, and when neither the back nor loins suffered pressure; and he tried a thousand different methods to prevent the miserable effects that all continued contact produced. In the remissions, of more or less duration, the patient had scarcely any fever, very little thirst, and partook with pleasure of light aliments. His dejections were always, or generally, very laborious, accompanied with wind, sourness, and often violent colics. The constipation was continued, and most obstinate. The patient could not make the necessary efforts for expulsion, but with difficulty, and in an ineffectual manner. The urine, scanty and loaded, was discharged with facility. Frequent erections still existed, and but a little time had elapsed since the patient had ceased to have connexion; the frequent want of which continued to be felt, with a remarkable degree of energy, almost to the day of his death.

A useless witness of these sufferings, I was not able to afford M. L. much relief. I lessened, by appropriate means, the phenomena of a gastro-intestinal irritation, which the tonic and antispasmodic medicines indiscreetly administered had brought on; but I could not retard the progress of the pulmonary consumption, into which he had fallen. This unhappy patient had a long and painful struggle, and at length yielded to the accumulated evils which overpowered him, on the 31st of October, at two o'clock in the morning, twenty-five days after I had been first called in to see him.

The body, opened thirty hours after death, by MM. Piedagnel and Lecouteux, in the presence of M. Majendie, who wished to assist me at this operation, was not sensibly altered, although it had remained in a warm situation. Its extenuation was most complete. The chest and upper extremities were particularly remarkable in this respect. The arms appeared as if glued to the body, and were, as during life, turned inwards. The legs and feet were slightly œdematous. The vertebral column, the particular object of our examination, presented in the upper half of the dorsal region, a slight curvature outwards and to the right side, and which raised the corresponding shoulder. The remainder of the back was well formed; the chest, very narrow, appeared still more so, in consequence of the elevation of the shoulders and the arms being brought forwards.

The adipose membrane had entirely disappeared. All the muscles were thin; those of the lumbar region were soft, and of a deep red colour. The psoas muscles were of the same hue, and they were nearly fluid: they were, however, neither inflamed nor suppurated; and we asked each other, how far this alteration in them accounted for those cruel pains which the patient had felt in the lumbar region, and especially on the right side. The brain was firm, very healthy, and contained a considerable quantity of serosity in the four ventricles; and this appeared to be transferible, according to the position of the body, into the cavity of the arachnoid lining the spinal canal: at least, we could not detect the existence of the cul de sac formed by this membrane, and which closes the fourth ventricle on this side. Nothing relative to the cerebellum appeared worthy of remark, nor could be connected with the prodigious generative power which had distinguished this patient.

The arachnoid membrane of the ventricles was very easily distinguishable; it appeared to be increased in thickness, but was not altered in transparency.

The canal of the spine was exposed along its whole extent, lifting up the spinous processes and the vertebræ: the marrow did not suffer any sort of compression; it was merely contorted with the spine itself in the dorsal region. The cavity of the arachnoid contained a considerable quantity of serum, beneath the part of this membrane united to the marrow; the proper membrane of this last was seen spread over with a great number of blood-vessels, arteries, and veins, highly injected with blood.

The spinal marrow, examined carefully in its situation and from behind, appeared at its upper part to be quite natural, from its origin to the fourth pair of cervical nerves. The two lower thirds, also, of its dorsal portion were in an equally sound condition; but, between these two portions, that is to say, for an extent of about six or seven inches, including the space of the two inferior thirds of the cervical portion and the upper third of the dorsal, inclusive, and corresponding to eight or nine pair of nerves, a most marked alteration was perceptible:—the marrow was of a softness so nearly approaching to fluidity, that the canal appeared to be filled with a real liquid, which was carried either upwards or downwards, according to the direction given to the dead body; but this fluid, which in both instances swelled the enveloping membrane of the spine, stopped precisely at those parts of that organ which preserved their natural condition. A little opening made in the dura mater *(of the spinal marrow)* gave issue to a great quantity of liquid. When that membrane was cut open, the spinal marrow was seen covered with its proper membrane: it was of a reddish grey colour, very soft; it exhibited a sensible fluctuation;* and, on opening its membrane, a liquid escaped, mixed with some little flakes of medullary matter. We afterwards opened this part of the cord largely by a longitudinal incision, which presented a long cavity, filled with a sort of reddish grey fluid, in which were spread a great number of capillary sanguineous vessels, of an extreme tenuity.† Upon the anterior part of this altered portion, the medullary cords were with difficulty seen in relation with the corresponding roots of the spinal nerves. On the left side, the interrupted cord, for the space of an inch and a half, was only marked by some lenticular portions of medullary matter, placed close to each other, in the line of its direction. This disposition appeared to us, probably, to have resulted from the escape of the matter which had taken place at this spot, in consequence of a small accidental opening made in the middle part of the membrane, or from the force employed upon the cords.

The marrow, detached and removed from its canal, may be examined on its anterior surface. Here the alteration which we have described

---

* The appearance of this part of the spinal marrow was such, that each of the assistants declared that there was a dropsy of the marrow itself. In fact, the colour natural to this part had disappeared, and was replaced by a colourless liquid, in which swam here and there some flakes of medullary matter.—M.

† For the first time I saw, as distinctly as possible, the minute cellular tissue of the spinal marrow: it was filled with the liquor of which M. Rullier speaks, but its laminæ and cells were most evident; they are even still more so in the portion which I have preserved in spirits.—M.

was a good deal less perceptible; the fluidity was not superficial, nor remarkable exteriorly; and the issue of the matter, in consequence of the incision that had been made, had diminished the volume, and removed all appearance of fluctuation. The medullary cords, corresponding to the origin of the threads of the anterior branches of the spinal nerves, were apparent, and did not show any interruption of their continuity, with the exception of the left side, which was altered as we have said. We traced them in the whole extent of the spinal cord, to the medullary tissue from whence they arise. An attentive dissection showed us that the disposition and structure of the original of the spinal marrow and its upper portion, down to the fourth pair of cervical nerves, presented nothing particular. Behind the inferior portion of the fourth ventricle, the posterior pyramids in front, the corpora pyramidalia, and olivaria, presented their usual configuration. In seeking carefully for the origin of the spinal nerve, we were convinced that the lower filaments of its origin corresponded evidently to the portion of the marrow that was destroyed.

The structure of all that part situated above the fourth pair of cervical nerves was sound; the medullary substance preserved its ordinary whiteness and consistence; but, below this point, this consistence and colour changed suddenly. It appeared as if the marrow was converted into a congeries of cells, *(une cellulosité)*, filled with a pale rose-coloured serum, as far as the sixth pair of cervical nerves; at which place there existed only a large cavity, whose limits were formed only by the vascular and serous membranes of the cord, and by the remains of the medullary matter. This disorganization was remarked as low as the fourth pair of dorsal nerves, and the alteration terminated in the form of a cone in the midst of the medullary substance, which appeared then with its usual properties.

The eight lower inches of this organ presented no alteration. Some nerves were dissected and pursued; those of the brachial plexus in particular, as corresponding to the disorganized part: they were found, as well as their ganglia, unaltered.

The lungs, apparently moist and crepitating in their anterior portions, were not sound; they adhered posteriorly to the pleura costalis, but in a slender manner. The left lung had some tubercles here and there in its superior lobe; the posterior border was loaded with blood. The right lung, more diseased, had the same appearance at the same part; it had the character of hepatization which follows chronic pneumonia, and exhibited several clusters of suppurated tubercles, some of the cysts of which were ossified.

In the abdomen, the stomach, considerably dilated, was sound; as were also the other viscera, with the exception of some portions of the small intestines, whose membranes, highly injected, were of a deep red colour, and slightly livid. Some blackish patches, apparent outwardly, corresponded with some small lenticular ulcerations of the mucous membrane. The bladder was small and thick, but sound.

*Remarks upon this Case,* by M. MAJENDIE.

To how many reflections do the preceding facts give rise? A man, enjoying, almost to his last hour, great moral activity, powerful generative faculties, a free movement of his inferior extremities, and the sensibility of the superior ones, had suffered, probably for a long time, the loss of one-third of the substance of the spinal marrow. The communication between the cervical and dorsal portion of this cord no longer existed, but by means of the membranes; for we have seen that there only remained a thin lamina of white substance, scarcely two lines broad, and most probably changed in its structure. The cavity from whence the medullary matter had disappeared, was filled with serosity. There was, then, nearly a complete isolation of the inferior from the superior part of the spinal marrow, and that for an extent of six or seven inches: nevertheless, the will exercised its power over the lower limbs, the imagination stimulated the genital organs, and those transmitted to the sentient being the lively emotions of pleasure. This persistency of communication of these two portions of the spinal marrow, is worthy of all our attention. We can deduce motives for some curious experiments. The great sympathetic nerve was not, certainly, the medium of communication; for all the sections or compressions of the spinal marrow intercept the determinations of the will relative to motion, and render those parts insensible that receive their nerves from that point of the marrow situated beneath the part compressed. There remains, then, the thin plate of medullary matter, and the proper membranes of the cord. If it is wished to prove that the lamina of medullary matter explains the difficulty, it would appear very extroardinary, considering its little extent in breadth and thickness, that it should perform this function as well as the marrow in its healthy state, and that there should not have been, at least, some diminution, either in the rapidity or the perfection of the transmission *(of the nervous energy).* If, on the contrary, it is thought that a medullary lamina so narrow is not sufficient for the explanation of the phenomena, it would be necessary to consider whether the vascular and serous membranes would not be fit for the nervous transmission. Nothing, it is true, hitherto has made this to be suspected; but nothing formally opposes itself to this explanation: it is a new and very important subject of research. This conjecture is the more to be permitted, because, in the subject of this observation, the membranes had preserved a perfect integrity.

With regard to the action of the heart, this case of M. Rullier is not less curious; for, according to the researches of Legalois, so great a diminution of the mass of the spinal marrow ought to have weakened the force of the contractions of the heart to such a degree, as to have prevented the blood from reaching to the lower extremities, and yet, notwithstanding, the circulation was carried on perfectly.

The contraction of the upper limbs, whilst at the same time they preserved their sensibility, deserves also to be noticed; for the posterior fasciculi of the spinal cord, the particular seat of sensibility, had disappeared as far as regards all those pairs of nerves composing the brachial plexus. Thus, the sensibility of the arms could not proceed from the ordinary source,—that is to say, that which corresponds to the posterior roots. There remain, then, (reasoning in accordance with my experiments,) the anterior roots, which alone afford motion, but are not entirely strangers to sensibility, as I have remarked before. Now, these roots were prolonged to the medullary lamina of communication; and, under this point of view, there will be no difficulty in the explanation, if one may be allowed to suppose, that which is not impossible, that there remained during life a small lamina towards the left side, as was found on the right. But a circumstance still more interesting, is that the anterior roots, as I have lately discovered in the preparation, had lost their medullary matter, and were reduced to their neurilema only, as it happens occasionally in the atrophy of the optic nerve. The posterior roots, on the contrary, had preserved their nervous matter to their junction with the membrane of the spinal cord. Every where else, except in this altered part of the marrow, the anterior and posterior roots presented equally the usual medullary matter.

This pathologic fact of the disposition of the anterior roots of the brachial nerves, is in perfect accordance with the complete immobility of the arms; but it is difficult to conceive that they were the agents of sensibility. Why, on the contrary, should not the posterior roots, which, far from being in a state of atrophy, were in their usual state, and consequently filled with medullary matter, have been the organs by which the arms of the patient preserved their sensibility, and suffered sometimes even acute pain?

This brings us again to conjecture that the immediate envelopes of the spinal marrow might be the conductors of sensibility, or even the sensible parts themselves. It has been seen, in the experiments that I have before related, that simple contact with the serous membrane of the marrow produces acute pain, if the posterior part of that organ is touched. I have lately proved this interesting fact upon the horse, in the presence of M. Dupuy.

The preparation shows very evidently that the internal cellular tissue of the spinal marrow, in the cells of which the medullary matter was probably lodged, established a solid connexion between the anterior and posterior parts of the cord: it was upon the laminæ of this tissue that the sanguineous vessels were ramified.

In conclusion, we shall repeat, that this case of M. Rollier's shows that we have still much to learn relative to the functions of the spinal marrow: and this ought to induce those persons who are engaged in pathologic anatomy to lose no occasion of examining that part, and even of preserving it when it exhibits any diseased appearance.

